# Patterning of Novel Breast Implant Surfaces by Enhancing Silicone Biocompatibility, Using Biomimetic Topographies

**Published:** 2010-04-26

**Authors:** S. Barr, E. Hill, A. Bayat

**Affiliations:** ^a^Plastic & Reconstructive Surgery Research, Manchester Interdisciplinary Biocentre, School of Translational Medicine, University of Manchester, Manchester, United Kingdom; ^b^Manchester Centre for Mesoscience & Nanotechnology, Information Technology Building, The University of Manchester, Oxford Road, Manchester, United Kingdom; ^c^Manchester Academic Health Science Centre, Department of Plastic and Reconstructive Surgery, University Hospital of South Manchester NHS Foundtion, Wythenshawe Hospital, Southmoor Road, Manchester, United Kingdom.

## Abstract

**Introduction and Aims:** Silicone biocompatibility is dictated by cell-surface interaction and its understanding is important in the field of implantation. The role of surface topography and its associated cellular morphology needs investigation to identify qualities that enhance silicone surface biocompatability. This study aims to create well-defined silicone topographies and examine how breast tissue–derived fibroblasts react and align to these surfaces. **Methods:** Photolithographic microelectronic techniques were modified to produce naturally inspired topographies in silicone, which were cultured with breast tissue–derived human fibroblasts. Using light, immunofluorescent and atomic force microscopy, the cytoskeletal reaction of fibroblasts to these silicone surfaces was investigated. **Results:** Numerous, well-defined micron-sized pillars, pores, grooves, and ridges were manufactured and characterized in medical grade silicone. Inimitable immunofluorescent microscopy represented in our high magnification images of vinculin, vimentin, and the actin cytoskeleton highlights the differences in fibroblast adhesion between fabricated silicone surfaces. These unique figures illustrate that fibroblast adhesion and the reactions these cells have to silicone can be manipulated to enhance biointegration between the implant and the breast tissue. An alteration of fibroblast phenotype was also observed, exhibiting the propensity of these surfaces to induce categorical remodeling of fibroblasts. **Conclusions:** This unique study shows that fibroblast reactions to silicone topographies can be tailored to induce physiological changes in cells. This paves the way for further research necessary to develop more biocompatible constructs capable of eliminating capsular contracture by subverting the foreign body response.

Breast augmentation is one of the most common cosmetic procedures in the developed world as well as being an effective reconstructive surgical option for women affected by breast cancer and congenital breast hypoplasia.^1^ Unfortunately, capsular contracture, which develops in a significant number of cases postimplantation, presents a clinical challenge because the capsule that develops around the implant can lead to pain, an undesirable cosmetic appearance, and patient dissatisfaction.^2^

Cell-cell and cell-substrate interactions play important roles in cellular biology and have strong and direct influences on body tissues and therefore on capsular contracture. The proposal that cells react to topographies was first demonstrated in 1964 by Curtis and Valde.^3^ Since then, cell adhesion and motility on artificial surfaces have been a central focus of research in the development of biomaterials.

Intuitively, it makes sense to emulate natural situations when designing synthetic products that will perform similar characteristics (to their natural contemporaries).^4^ This natural inspiration served as a basis for our surface production. To produce a surface with a scientific pedigree, structured research is required, which works from first principles and quantifies the reaction of body tissue to simple surface features. Ideally, an implant surface should be capable of evading host recognition or attack and of reducing the foreign body reaction that is often induced by biomaterials.

The aim of this study is therefore to look at how breast-derived human fibroblasts react and align to different well-defined micron-patterned silicone surfaces in vitro, an area of research that has been deficient since the inception of the first breast implant in 1964.^5^

## MATERIALS AND METHODS

The methods we used to achieve this involved the following:

1. Creating new topographies in polydimethylsiloxane (PDMS)
2. Quantifying these PDMS surfaces using microscopy
3. Culturing breast-derived human fibroblasts on these PDMS surfaces
4. Evaluating the effect of these silicone surfaces on breast-derived fibroblasts

The methods of manufacturing the surfaces created in this study are derived from a series of elemental considerations that are important when large-scale manufacturing is considered.

## Creating new topographies in PDMS

We manufactured a range of molds with different surface features, before using these as templates to form medical grade PDMS surfaces.

MED-6215 (NuSil, Carpinteria, California) optically clear elastomer was the PDMS chosen for this study. The product profile matched closely the tensile strength and coefficients of the silicones used in breast implants currently available.^6^

**Figure 1:**
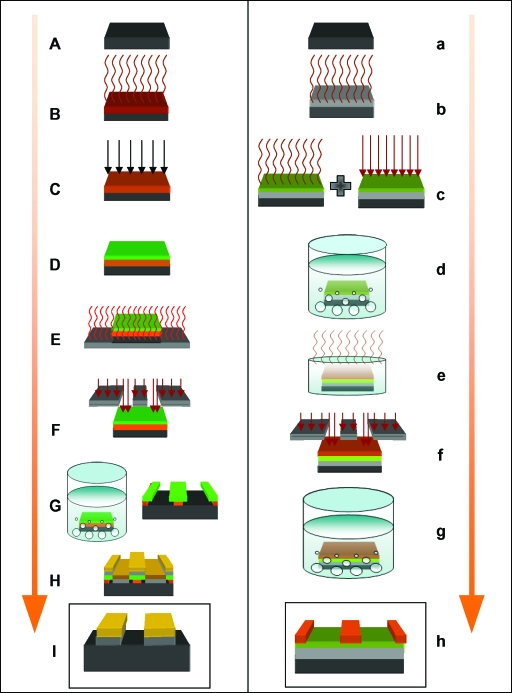
Flow diagram of the processes used to produce S1805 and the SU-8 surfaces. S1805 resist protocol: (A) Clean 22 mm × 25 mm silicon wafer (black); (B) 1 mm^3^ of HMDS (brown) applied to the wafer and spun at 3000 rpm for 45 seconds; (C) 1 mm^3^ of PMGI (orange) applied to the wafer, spun at 3000 rpm for 45 seconds and ultraviolet, and exposed for 10 seconds; (D) 1 mm^3^ of S1805 (green) applied to the wafer and spun at 4000 rpm for 30 seconds; (E) baked. at 110°C for 1 minute; (F) expose the resist to ultraviolet light using the Karl Suss mask aligner for 10 seconds; (G) develop pattern in MF319 Alkali for 50 seconds and rinse in DI water to produce pattern; (H) deposit 5 nm of chromium (grey) and 20 nm of gold (gold) in the Moorfield Deposition System and perform liftoff in 1165; and (I) final product, showing surface features in chromium and gold. SU-8 Resist protocol: (a) Clean 22 mm × 25 mm silicon wafer (black); (b) deposit 5 nm of chromium (grey) using the Moorfield Deposition System followed by HMDS primer; (c) SU-8 2000.5 (green) applied to the wafer and spun at 3000 rpm for 45 seconds followed by 95°C bake and ultraviolet light exposure for 4 seconds; (d) develop for 1 minute in EC solvent; (e) deposit SU-8 2025 (orange) onto wafer at 4000 rpm return to hotplate and ramp bake within an upturned petri dish at 95°C for 10 minutes; (f) expose SU-8 2025 resist for 10 seconds and return to 95°C hotplate for 5 minutes; (g) develop in EC solvent for 5 minutes; and (h) final product patterned SU-8 2025 wafer.

Molds were produced using photolithography, a process capable of fabricating large areas of patterned substrate in chemical materials, known as photoresists. We developed 2 complex protocols (Fig 1) to generate features in 2 types of photoresist. These photoresists were chosen as they provided us with 2 contrasting feature heights in silicone of 0.5 µm and 20 µm, which also fell within the working range of our equipment. These photoresists were the S1805 Shipley (Coventry, UK) photoresist and the SU-8 2025 Microchem (Newton, Massachusetts) photoresist.

**Figure 2:**
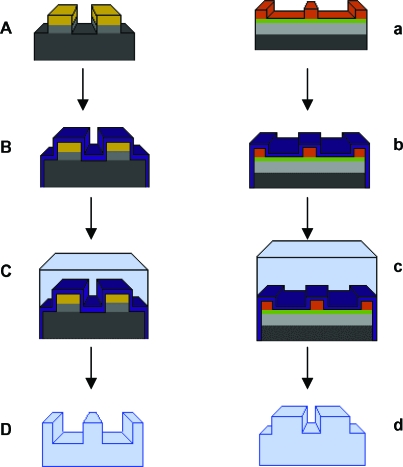
Surface fabrication. (A) Molds produced using photolithography as explained in Figure 1 are (B) coated in a 5-nm-thick layer of PMMA (PURPLE) and allowed to dry (C) before a layer of PMDS is spun onto the surface, baked at 150°C for 30 minutes, and peeled to produce a (D) PDMS imprint of the original mold.

Before spinning PDMS onto the surface of these substrates, each mold surface (demonstrated in box I of the S1085 column and box h of the SU-8 column of Fig 1) required a releasing agent to be able to peel the PDMS. We used poly(methyl methacrylate) (ALLRESIST GmbH, Strausberg, Germany), which we mixed at a concentration of 1.5% and span this releasing agent onto the mold surfaces at 3000 rpm, which produced a layer of poly(methyl methacrylate), 5-nm thick over the surface of the mold (Fig 2).

Polydimethylsiloxane was then spun onto the surface of these wafers at 700 rpm, which corresponded to a PDMS thickness of approximately 40 µm at the central portion of the mold. The PDMS was hardened or “cured” at 150°C for half an hour, allowed to cool and peeled from the surface of the mold, floated on distilled water, and allowed to settle onto the surface of a clean glass cover slip. In total, thirty-two 0.5-µm-high surfaces and three 20-µm-high surfaces were fabricated in a range of pillars, pits, and ridges.

## Surface characterization

Polydimethylsiloxane surfaces produced using the protocols were imaged and their surface characteristics measured using the FEI XL30 Sirion FEG Scanning Electron Microscope (Hillsboro, Oregon), a Nikon eclipse LV100POL light microscope (Tokyo, Japan), and a ThermoMicroscopes Autoprobe CP Research System (California) Atomic Force Microscope.

## Cell culture

Fibroblasts produce the collagen that “walls off” the implant from the surrounding tissue and encapsulates the breast implant.^7^ Therefore, in this study, primary culture fibroblasts derived from normal breast tissue were used and cultured from a control patient with no history of fibrosis or abnormal raised dermal scarring such as hypertrophic or keloid scarring.

Fibroblasts were grown initially in 75-mL culture flasks in Dulbecco's Culture Medium (DMEM) substituted with 10% fetal bovine serum, 1% nonessential amino acids (Lonza, Basel, Switzerland), and 1% Pen Strep (Lonza). Media was replaced every 2 days during culture. Fibroblasts were grown in an incubator at 37°C with a 5% CO_2_ atmosphere.

Polydimethylsiloxane surfaces were first sterilized in ethanol for 5 minutes and allowed to air dry in a Petri dish within the culture hood before seeding with fibroblasts. Fibroblasts were trypsinized with trypsin EDTA (Lonza) for 5 minutes and spun down at 1500 rpm in a centrifuge, and the supernatant was poured off before being resuspended in media. PDMS surfaces are highly hydrophobic; therefore, we used fibronectin, a provisional protein matrix protein as a coating agent for the surfaces we created. We submerged our surfaces in a 5 µg/mL concentration of fibronectin (Sigma, Dorset, UK) in PBS for 1 hour at 37°C before rinsing once again in PBS, which increased surface wettability. Cells were seeded onto the surface of the fibronectin-coated PDMS at a concentration of 1 ×10^5^ cells per milliliter of media, which was achieved by using a C-Chip disposable hemocytometer (Labtech, Ringmer, East Sussex).

## Evaluating the effect of PDMS topographies

Microscopy was performed on a Leica (Wetzlar, Germany), TCS SP5 AOBS inverted confocal microscope with a Leica (Wetzlar, Germany), DMI6000 inverted microscope body, and an external Motorised XY stage. A 20×/0.70 multi-immersion HC PL Apo lens, a 40×/0.75 oil immersion HCX PL Fluotar lens, and a 63×/0.60-1.40 oil immersion HCX PL Apo lens (Zeiss, Jena, Germany) were used.

The cytoskeletal components vinculin, vimentin, and actin were all stained to illustrate the effects of surface topography on the cytoskeletal dynamics: vinculin, using an anti-vinculin antibody (Sigma Monoclonal Anti-Vinculin antibody produced in mouse clone hVIN-1 V9131); vimentin, using a monoclonal anti-vimentin antibody (Vector Laboratories, Peterborough, UK, VP-V683) and actin, using Rhodamine Phalloidin (Universal Biologies, Cambridge, UK). The nucleus was also highlighted using 4',6-diamidino-2-phenylindole, dihydrochloride (DAPI) Sigma (Dorset, UK). Results were exported to Image-J 1.41 (National Institutes of Health Bethesda, Maryland)^8^ and combined using the color functions within the MacBiophotonics software plugin (Hamilton, Ontario, Canada).

## RESULTS

### Surface creation and characterization

**Figure 3:**
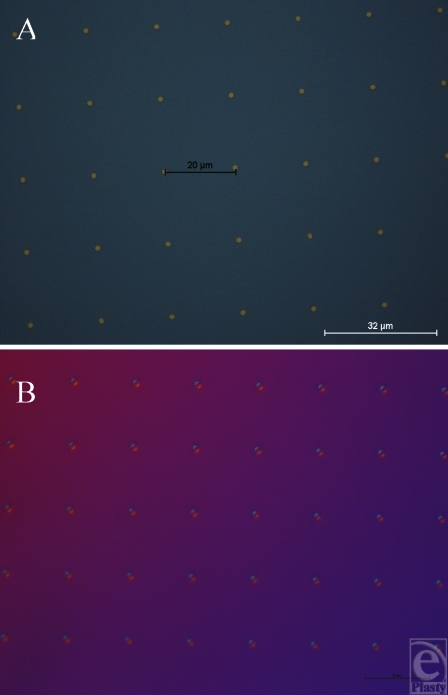
S1805 photoresist mold surfaces (0.5-µm height). (A) Gold pillars of 4-µm width with 18-µm spacing. (B) Silicone impression produced using our peeling protocol taken from the mold in A to produce 4-µm-wide pits with 18-µm spacing.

**Figure 4:**
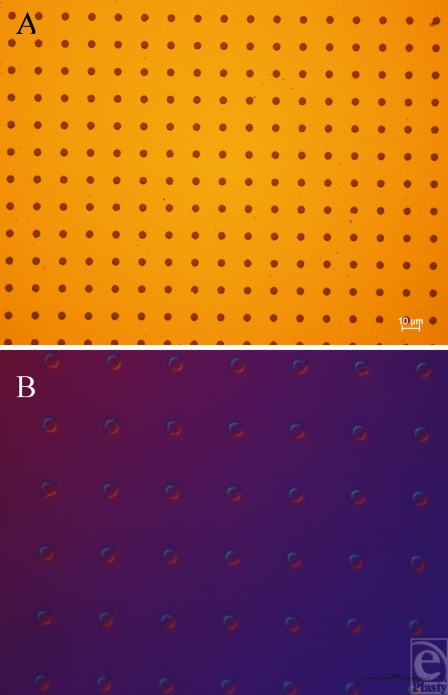
S1805 photoresist mold surfaces (0.5-µm height). (A) Pits of 4-µm wide with 13-µm spacing in gold. (B) Silicone impression taken from the mold in A using our peeling protocol to produce 4-µm-wide pillars with 13-µm spacing.

**Figure 5:**
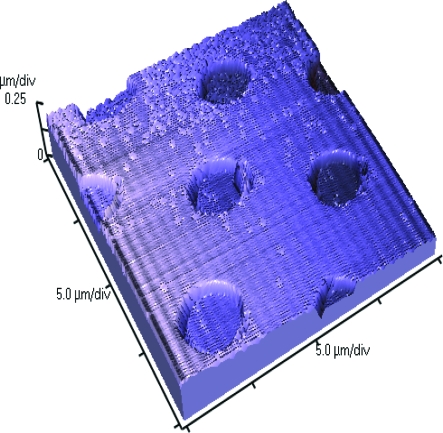
Atomic force microscope image of 4-µm-wide and 5-µm-spaced pits in silicone that illustrates how well the features were transferred to silicone.

We created a range of 32 different topographies in PDMS, using the S1805 techniques described in the “Methods” section as well as a smooth control for comparison (Figs 3 and 4) (Table 1). Atomic force microscopy scanning of our silicone surfaces and their corresponding molds showed that the mold transferred well to our silicone surfaces and also that the sidewalls of the features of the imprinted surface were vertical and well-defined (Fig 5).

**Figure 6:**
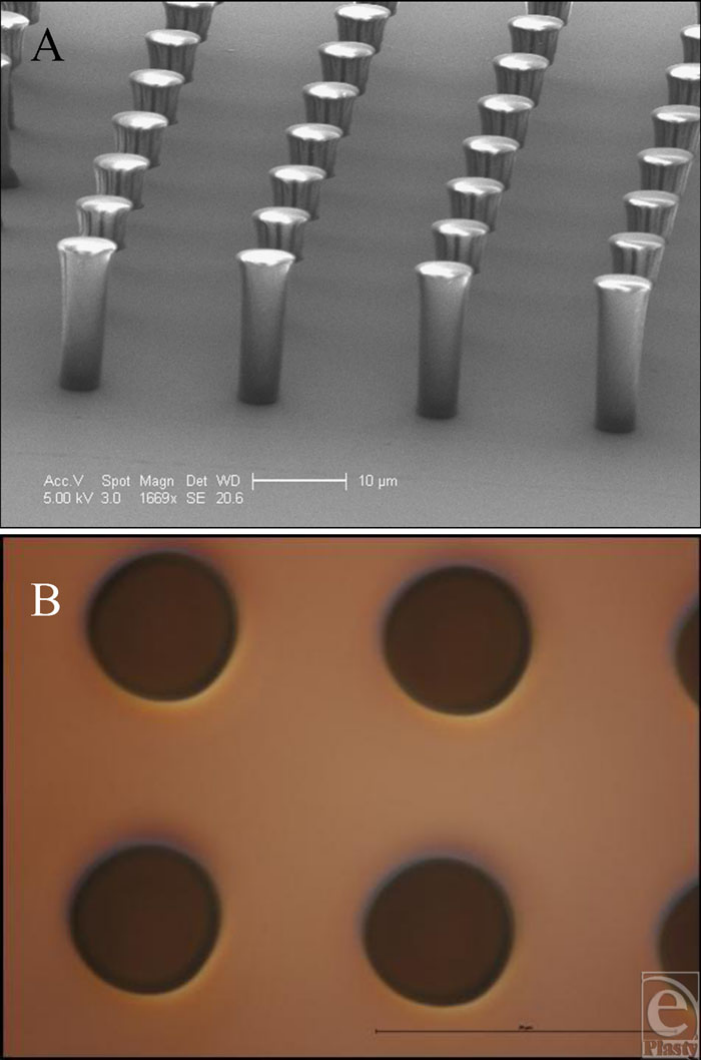
Features produced in SU-8. (A) Electron microscope image of the 5-µm-wide and 15-µm-spaced pillars produced using our SU-8 protocol. (B) 5-µm-wide and 15-µm-spaced pits produced in silicone as an imprint of surface A.

**Figure 7:**
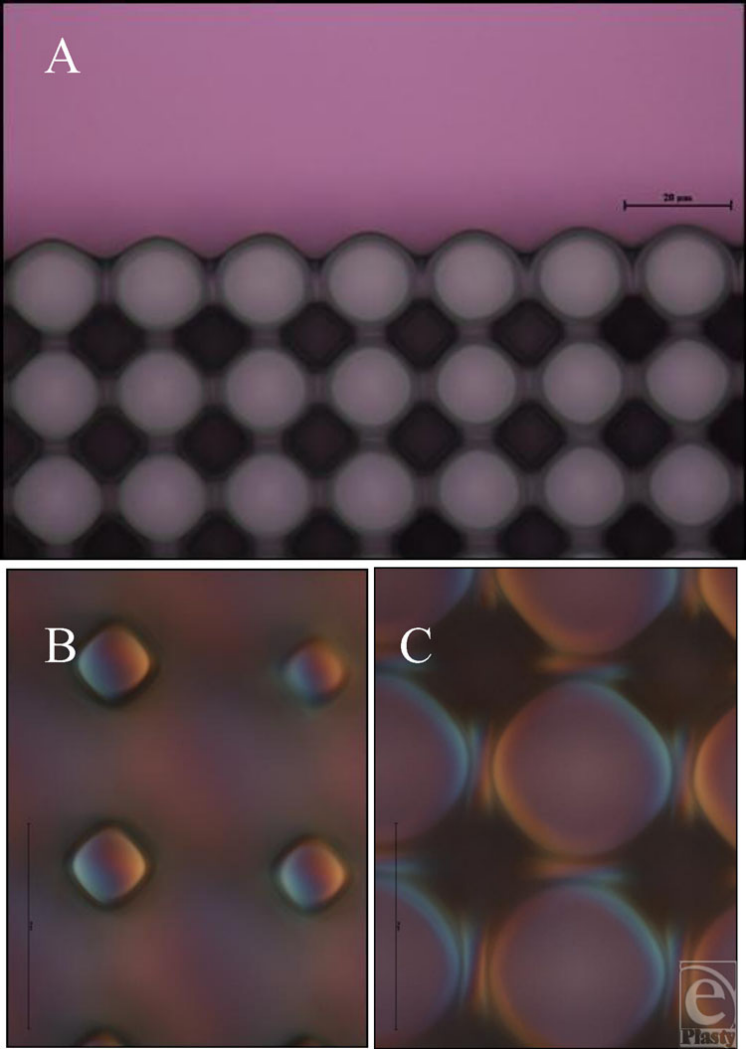
Features produced in SU-8. (A) Light microscope image of the 10-µm-wide, 20-µm-spaced pits produced in silicone adjoining an area with no features upon its surface. (B) 10-µm-wide, 20-µm-pillar upper surface produced in silicone as an imprint of the mold in A and (C) the lower surface of these features.

Manufacturing using these techniques therefore produces consistent results and could reasonably be performed to produce features in a commercial implant. PDMS has been shown to offer good repeatability and peeling on other substrates down to approximately 100-nm-sized features, using a form of imprint molding described by Koo et al^9^ and Schmid and Michel.^10^ Smaller-sized features in PDMS are also theoretically possible using similar technologies to the ones developed in this study. The only restriction to this is the resolution achievable using photolithography, approximately 1 µm. We produced several SU-8 surfaces with 20-µm heights (Figs 6 and 7) (Table 1), including: 100-µm lines, 10-µm wide and 20-µm spaced pits, and 5-µm wide and 15-µm spaced pillars, which were successfully transferred to PDMS.

## Cell culture results

### Smooth controls

**Figure 8:**
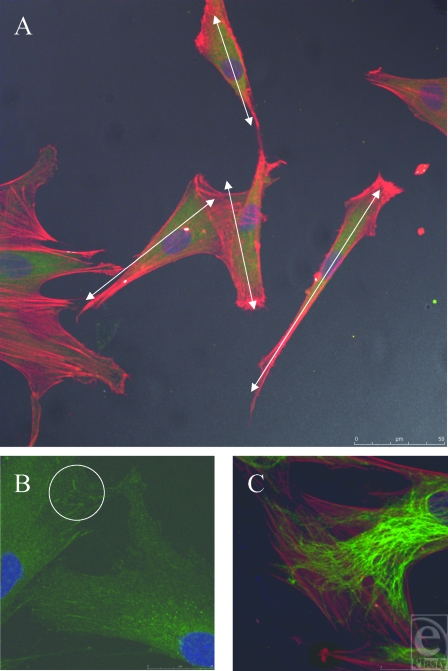
Smooth control surface. (A) Fibroblasts randomly align in multiple directions. White arrows show differing polarities of fibroblasts. Actin fibers (red) and vimentin (green). (B) Vinculin (green) stained focal adhesion plaques highlighted in white circle, illustrating the point of contact between fibroblast and underlying surface. (C) Actin filaments and intermediate fibers have different directions within one cell. Actin fibers (red) and vimentin (green).

Smooth controls caused the fibroblasts to spread randomly across its surface. No directional regularity was observed, which is typical of fibroblasts (Fig 8A). This is indicated by the white arrows in Figure 8A, which demonstrate the nonuniform polarity of the cells. Cells on the smooth control also vary in their dimensions, some having a longer thinner cell body with bunching of their actin fibers, whereas others are more nucleated, with fanned actin fibers stretching in different directions.

Higher magnification reveals the focal adhesions, demonstrated as condensations of vinculin staining, which the cell uses to anchor itself to the substrate it is in contact with (Fig 8B). Intermediate fibers have a less directionally organized arrangement within the cell and an indefinite appearance. Intermediate fibers do not necessarily run parallel with the direction of the actin stress fibers (Fig 8C).

## S1805 resist–derived surfaces (0.5-µm-high features)

**Figure 9:**
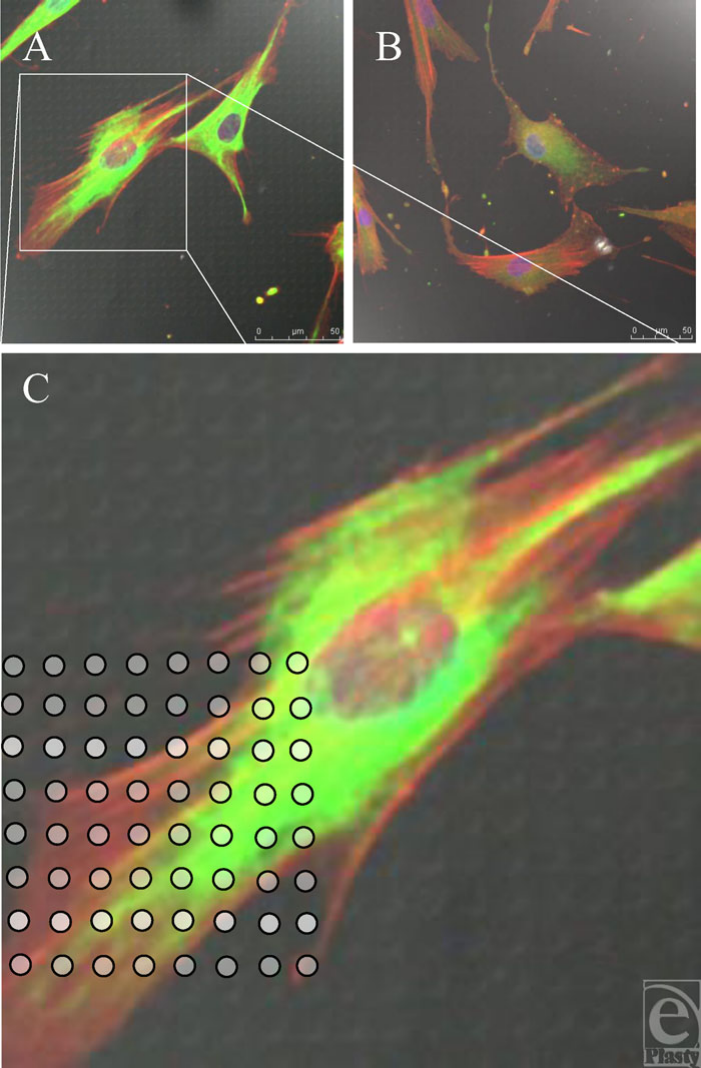
S1805-derived surfaces. (A and B) Cells randomly spread upon this surface. (C) White dots (indicating surface features beneath the fibroblast) highlight the lack of increased staining of fibroblast cytoskeleton in relation to surface features. Actin fibers (red) and vimentin (green).

Cells cultured on these surfaces did not exhibit distinctive cytoskeletal changes compared to smooth controls (Fig 9). Cells were not drawn toward adhering to the pillars or pits on these surfaces. If cells react differently upon these surfaces compared with smooth controls, one would expect an increase, decrease, or absence of staining for vinculin, vimentin, or actin within the superimposed circles in Figure 9C. However, there was no evident change in staining related to these features.

## SU-8 resist–derived surfaces (20-µm-high features)

**Figure 10:**
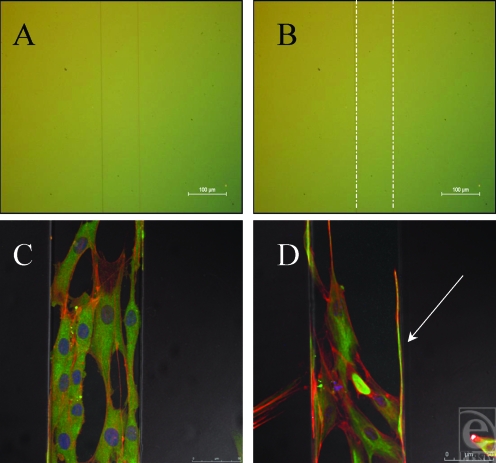
Fibroblasts align to the 100-µm ridge topography. (A) 100-µm ridge topography in silicone. (B) 100-µm ridge topography highlighted with white lines. (C) The cells along the surface of this feature are unable to reach down from this feature and as such grow along it. (D) Fibroblasts send filopodia along the edge of this feature (highlighted by the white arrow within this figure), searching along is edge, a classic example of contact guidance. Actin fibers (red) and vimentin (green).

**Figure 11:**
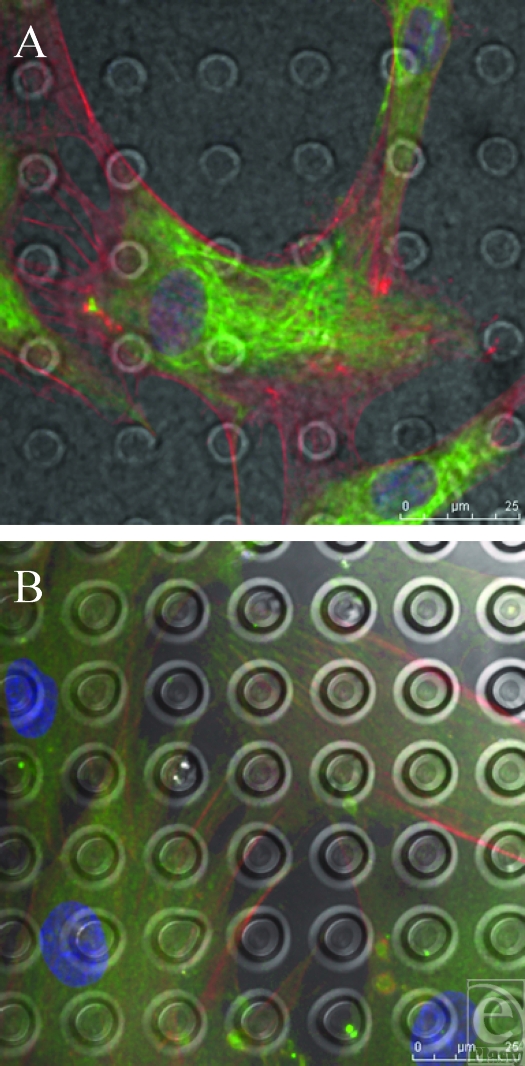
SU-8-derived, 5-µm-wide, 15-µm-spaced pits. (A) and (B) Pit surface produced from the SU-8 master has had little effect on the morphology of the fibroblasts upon its surface as no concentration of any of the actin, vimentin, or vinculin staining upon its surface is observed in relation to features beneath the fibroblasts. Actin fibers (red) and vimentin (green).

**Figure 12:**
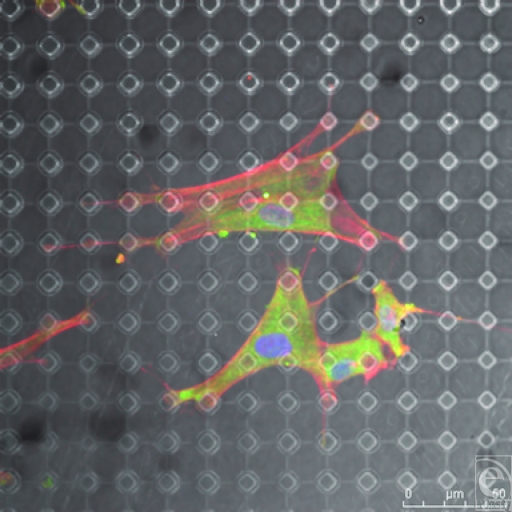
SU-8-derived, 10-µm-wide, 20-µm-spaced pillars. Fibroblasts send projections to surrounding pillars for support. The pillared surface has altered the shape of these cells from those illustrated in Figure 8. Actin fibers (red) and vimentin (green).

**Figure 13:**
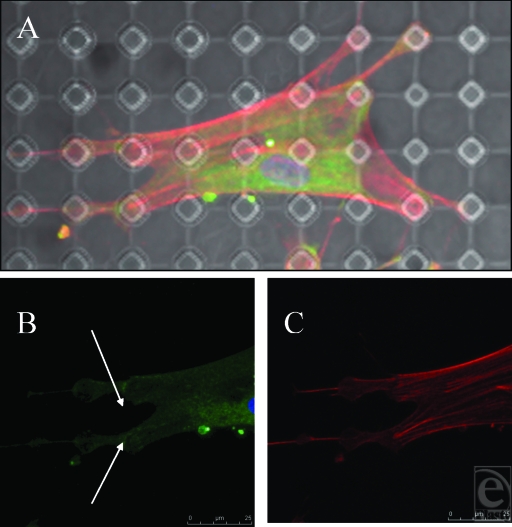
SU-8-derived, 10-µm-wide, 20-µm-spaced pillars (B) and (C) show higher magnification of the cell contained in (A). Actin fibers (red) run from a focus at the top of the pillars, whereas the vinculin (green) runs from the edge of these pillars.

**Figure 14:**
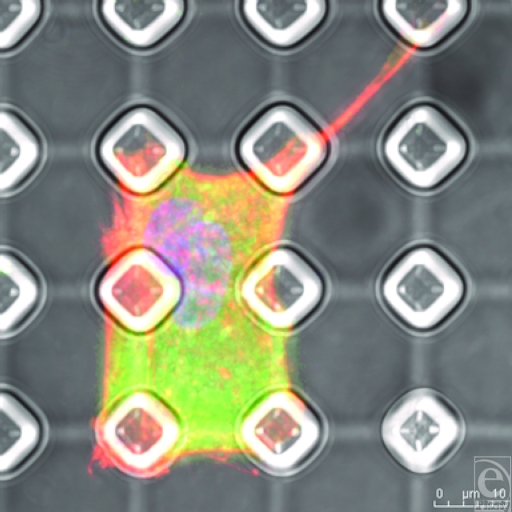
SU-8-derived, 10-µm-wide, 20-µm-spaced pillars. Conformational change is elicited by the surface with which the cell is in contact. The cell in this figure is isolated within a complex of 6 pillars and has become square in shape because of this but still sends out a protrusion.

**Figure 15:**
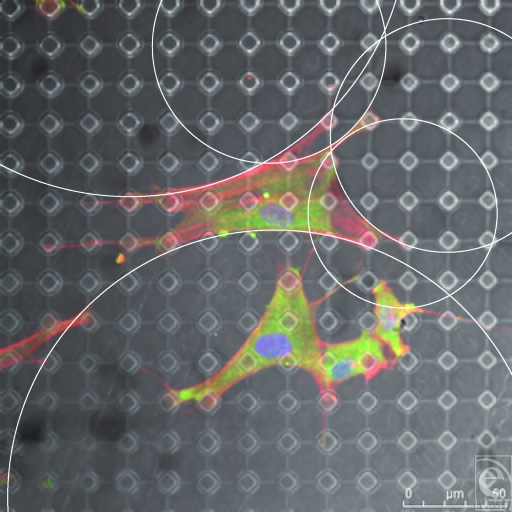
SU-8-derived, 10-µm-wide, 20-µm-spaced pillars. Each cellular connection is defined by an arc from one point of adhesion to the next with the arc being defined by the distance between these 2 points. This is illustrated by the white circles within this figure. Actin fibers (red) and vimentin (green).

**Figure 16:**
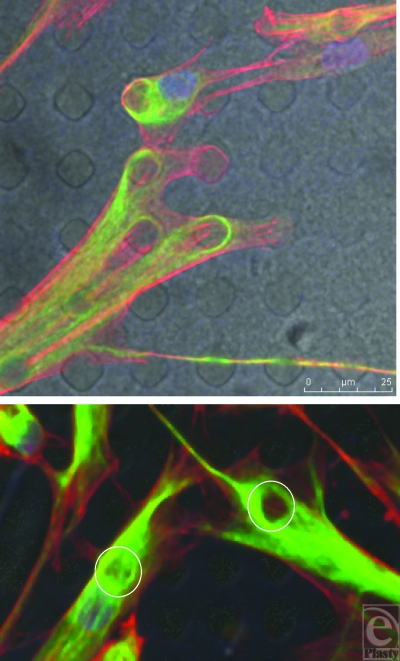
SU-8-derived, 10-µm-wide, 20-µm-spaced pillars. Vimentin (green) staining above the pillar arrays beneath the cell is reduced. Intermediate fibers are therefore deficient above cellular attachment points highlighted by the white circles in Figure B. Actin fibers (red).

SU-8 surfaces provoked dramatic changes in fibroblast conformation. 100 µm wide ridges caused an alignment of the cells to this feature; 5-µm-wide, 15-µm-spaced pits did not produce a noticeable effect in cytoskeletal organization whereas 10-µm wide, 20-µm-spaced pillars produced a distinctive effect on fibroblast morphology (Figs 10–16).

## DISCUSSION

This study shows that numerous, well-defined micron-sized pillars, pores, grooves, and ridges were manufactured and characterized in medical grade silicone. Inimitable immunofluorescent microscopy represented in our high magnification images of vinculin, vimentin, and the actin cytoskeleton highlights the differences in fibroblast adhesion between fabricated silicone surfaces. These unique figures illustrate that fibroblast adhesion and the reactions these cells have to silicone can be manipulated to enhance biointegration between the implant and the breast tissue. An alteration of fibroblast phenotype was also observed, exhibiting the propensity of these surfaces to induce categorical remodeling of fibroblasts.

## S1805-created surfaces

Fibroblasts did not appear to detect the surface features of the S1805-derived features (Fig 9). Other studies into the limits of cell sensing have looked into filopodia, the cytoplasmic cell sensing organelles that extend from the leading edge of a migrating cell.^11^ A study into their ability to identify the limit of their sensory abilities has shown that they are, however, capable of sensing features down to 10 nm in polymer demixed surfaces.^12^ It is probable that the cells are in fact sensing the S1805 PDMS surfaces imaged; however, the techniques used in this study do not illustrate this interaction.

## SU-8-derived, 100-µm-raised ridges

Contact guidance, the directional alignment of cells has been previously described^13^ and microgrooves and ridges are some of the most extensively studied surfaces due to their ease of manufacturing and the ease in which it can be established whether a cell has reacted and aligned to these topographies.^14^ The surfaces created in this study induced a linear direction in fibroblasts in contact with this surface (Fig 10). When microgrooves showed a periodicity greater than 2 µm, cells aligned to these surfaces due to the alignment of the cytoskeletal elements and the direction of the focal adhesions.^15^ As the cell only comes in contact with one edge of the pattern and does not span 2 or more features, it is therefore likely that the cell has recognized this pattern, not as a ridge, but as a “step pattern.”^16^

The fibroblasts have been deflected along the surface of this ridge in Figure 10, not by contact guidance brought about by the alignment of the focal adhesions, but by the sharp drop present on either side of the feature. The sudden absence of a substrate beneath these sensory organelles causes the fibroblast to retract and follow a new direction. The white arrow in Figure 10D denotes a long filamentous cellular protrusion that has clearly sensed its way along the edge of this feature.

This surface has therefore induced some contact guidance of the cells present on its surface; however; to truly induce a controlled orientation, where all the cells run in identical directions, a smaller period is required. A groove width of 5 µm or less has been shown to be optimum for fibroblast orientation on other surfaces. Clark et al^15^ postulated that this was due to these 5-µm substructures resembling the fibrillar extracellular matrix that provides supporting structure to all cells throughout the body.^17^,^18^

## 10-µm-wide, 12-µm-spaced pits

The reaction to this surface may be due to a flow in the resist that had not been sufficiently cured, and as the PDMS surface was an imprint of the SU-8 surface, this reflow would have affected the top surface of the topography, thus rounding off the side walls of the pits. It has been shown that pit edges have a direct effect on cell reactions to topographies and that cells often use pit edges as “foot-holds,” it is possible that the absence or reduction in these defined features may have altered the way in which fibroblasts reacted to them.^19^ This would have reduced the reaction of the cells to any definite edge that would normally have provoked an increased uptake of anti-vinculin and a bright area of fluorescence corresponding to this adhesion plaque.

## 10-µm-wide, 20-µm-spaced pillars

This surface has had a notable effect on the shape of the fibroblasts as shown from Figure 12. These surfaces promote a range of effects on the different cytoskeletal components. Focal adhesions condensed around the tops of the pillars, drawn to the stability of the only structures available to them on this surface (Fig 13B). These condensations are most focused around the edge of the structures. As the actin fibers pull these processes across the body of the cell, the largest focal adhesions form at the points of maximum tension between the cell and its surface. Rather like a tent, the cell looks to anchor itself to the substrate using a series of guy rope–like protrusions stretching itself across the surface like a canvas to give itself maximum adhesion and drawing the surface topography toward the center of the cell.^20^ Fibroblasts exhibit a shape that it determined by spanning their adhesive connections to the underlying substrate. These connections form arcs between these structures as shown from Figure 15 and the shapes of these arcs are determined by the distance between these structures as a derivative of La Places law.^21^ Actin fibers run from these pillars to adjoining pillars on the surface of the PDMS and are most densely stained between these structures because of the stability the pillars provide the cell (Fig 13C).

It has been shown that cells that bunch up and become more nucleated often undergo a form of cell death known as anoikosis.^22^ This is not universally apparent on this surface and the cells are well spread. On some occasions, cells do seem to struggle with the topography, only covering several of the pillars and adopting a very square cell shape, prescribed by the topography. However, as shown in Figure 14, the cells send out filopodia and lamellae to survey the surface around them. Staining for the vimentin reveals a different pattern to the way the intermediate fibers react to the surface. These fibers, unlike the focal adhesions, seem more affected by topographies beneath the cell rather than at its periphery. Intermediate fibers seem to become deficient in areas directly above these pillars (Fig 16).

Intermediate fibers act as a cell organelle and nuclear support and also keep the shape of the cell through its connection with the cell membrane. These pillars reduce support to the cell body overlying these structures leaving a punched-out structure to the intermediate fiber cytoskeleton. It has additionally been shown by Helfand et al^23^ that vimentin and also by Goldman and Ingber^24^ that vinculin both participate in signal transduction, up- and downregulating gene expression. Myofibroblasts have been implicated in the development of capsular contracture and as such their presence on the surface of the implants created is important as the fibroblasts seeded on to these surfaces were initially fibroblasts (not myofibroblasts).^25^ The simple definition of a myofibroblast, “smooth-muscle–like fibroblasts,” would suggest that the surfaces above have induced a conformational change in the cells and caused them to become myofibroblast-like.^26^^-^27. However, the exact role of the myofibroblast in capsular contracture and its implant surface interaction would require further investigation which is beyond the scope of this paper and its preliminary findings.

## CONCLUSIONS

The results presented in this article have illustrated that the creation of silicone surfaces with quantifiable topographies is achievable and that these topographies are capable of inducing directional control of cell growth and alterations in cell shape and phenotype. The protocols developed in this study are capable of producing endless variations of topographies in PDMS. This represents the foundations for the next stage of advanced development of silicone biomaterials for novel breast implants.

## Figures and Tables

**Table 1:** Features created in silicone^a^

S1805 resist–created surfaces (0.5-µm feature height)			
2-µm pillars, 18-µm spacing	2-µm pits, 18-µm spacing	4-µm pillars, 18-µm spacing	4-µm pits, 18-µm spacing
2-µm pillars, 13-µm spacing	2-µm pits, 13-µm spacing	4-µm pillars, 13-µm spacing	4-µm pits, 13-µm spacing
2-µm pillars, 8-µm spacing	2-µm pits, 8-µm spacing	4-µm pillars, 8-µm spacing	4-µm pits, 8-µm spacing
2-µm pillars, 7-µm spacing	2-µm pits, 7-µm spacing	4-µm pillars, 7-µm spacing	4-µm pits, 7-µm spacing
2-µm pillars, 6-µm spacing	2-µm pits, 6-µm spacing	4-µm pillars, 6-µm spacing	4-µm pits, 6-µm spacing
2-µm pillars, 5-µm spacing	2-µm pits, 5-µm spacing	4-µm pillars, 5-µm spacing	4-µm pits, 5-µm spacing
2-µm pillars, 4-µm spacing	2-µm pits, 4-µm spacing	4-µm pillars, 4-µm spacing	4-µm pits, 4-µm spacing
2-µm pillars, 3-µm spacing	2-µm pits, 3-µm spacing	4-µm pillars, 3-µm spacing	4-µm pits, 3-µm spacing
	**SU-8-created surfaces (20-µm feature height)**		
10-µm pits 20-µm spacing	5-µm pillars 15-µm spacing	100-µm-wide ridges	

^a^The surfaces we created in silicone, using both the SU-8 and S1805 protocols, are described in the “Methods” section.
